# Fluoride Content of Ready-to-Eat Infant Foods and Drinks in Australia

**DOI:** 10.3390/ijerph192114087

**Published:** 2022-10-28

**Authors:** Navira Chandio, James Rufus John, Shaan Floyd, Emily Gibson, Danny K. Y. Wong, Steven M. Levy, Judy R. Heilman, Amit Arora

**Affiliations:** 1 Campbelltown Campus, School of Health Sciences, Western Sydney University, Locked Bag 1797, Penrith, NSW 2751, Australia; 2 Health Equity Laboratory, Campbelltown, Sydney, NSW 2560, Australia; 3 Translational Health Research Institute, Western Sydney University, Locked Bag 1797, Penrith, NSW 2751, Australia; 4 School of Psychiatry, University of New South Wales, Sydney, NSW 2052, Australia; 5 South Western Sydney Local Health District, Liverpool, Sydney, NSW 2170, Australia; 6 Ingham Institute of Applied Medical Research, Liverpool, Sydney, NSW 2170, Australia; 7 School of Natural Sciences, Macquarie University, Sydney, NSW 2109, Australia; 8 College of Dentistry and Dental Clinics, University of Iowa, Iowa City, IA 52242, USA; 9 Discipline of Child and Adolescent Health, Sydney Medical School, Faculty of Medicine and Health, The University of Sydney, Westmead, Sydney, NSW 2145, Australia; 10 Oral Health Services, Sydney Local Health District and Sydney Dental Hospital, NSW Health, Surry Hills, Sydney, NSW 2010, Australia

**Keywords:** fluoride, dental fluorosis, infant formula, infants, infant foods, baby milk

## Abstract

The use of fluoride is effective in preventing dental caries. However, an excessive intake of fluoride leads to dental fluorosis, making it necessary to regularly monitor the fluoride intake especially for infants. There is hitherto a lack of information on fluoride content in infant foods from an Australian perspective. Therefore, this study aims to estimate the amount of fluoride content from a range of commercially available ready-to-eat (RTE) infant foods and drinks available in Australia. Based on an external calibration method, potentiometry involving a fluoride ion selective electrode and a silver|silver chloride reference electrode was conducted to analyse the fluoride content of a total of 326 solid food samples and 49 liquid food samples in this work. Our results showed an overall median (range) fluoride content of 0.16 (0.001–2.8) µg F/g of solid food samples, and 0.020 (0.002–1.2) µg F/mL of liquid food samples. In addition, ~77.5% of the liquid samples revealed a fluoride content < 0.05% µg F/mL. The highest variation of fluoride concentration (0.014–0.92 µg F/g) was found in formulas for ≥6 month-old infants. We have attributed the wide fluoride content variations in ready-to-eat infant foods and drinks to the processing steps, different ingredients and their origins, including water. In general, we found the fluoride content in most of the collected samples from Australian markets to be high and may therefore carry a risk of dental fluorosis. These results highlight the need for parents to receive appropriate information on the fluoride content of ready-to-eat infant food and drinks.

## 1. Introduction

The beneficial role of fluoride in preventing dental caries and promoting optimal oral health is well established worldwide [1,2]. The introduction of fluoride to community water systems has been widely recognised as one of the public health measures in disease prevention owing to the significant reduction in caries prevalence in most industrialised nations [3,4]. Studies indicate that optimum levels of fluoride from various sources such as drinking water, toothpastes, and diet reduce tooth caries through several mechanisms such as inhibition of plaque formation by bacterial enzymes, subsequently inhibiting demineralisation, and also enhancing remineralisation [5,6]. There is evidence suggesting that subjecting one to optimum fluoride levels during infancy and early childhood years is critical not only for tooth development, but is also associated with less incidence of dental caries in adulthood [7].

Whilst fluoride intake can be beneficial in reducing caries experience, ingestion of moderate levels of fluoride (1.5–4.6 ppm) can lead to a detrimental condition called dental fluorosis in children [8,9]. Dental fluorosis is characterised by several dental aberrations ranging from aesthetically unappealing white or brown staining in mild forms to pitting and mottling of tooth surfaces in severe cases [10]. Although the prevalence of dental fluorosis has decreased over the years, the National Health and Medical Research Council of Australia reports that water fluoridation at the current levels (0.6 to 1.1 mg/L) may be associated with mild forms of dental fluorosis [11]. In addition, there are studies reporting higher than expected prevalence of dental fluorosis in Australia [12,13,14].

As infants in their tooth enamel formation years are highly susceptible to dental fluorosis, it is imperative to have systems in place to continuously monitor the fluoride concentration to effectively manage this preventable condition. There is evidence suggesting that the first 20 to 30 months of infanthood is a critical period for fluoride overexposure; therefore, it is imperative to closely monitor fluoride intake from as many available sources as possible [10]. There is general consensus that a daily fluoride intake of 0.05 to 0.07 mg/kg is considered optimal for oral health benefits [15,16]. Whilst numerous studies report on the prevalence of dental fluorosis attributed only to fluoride levels in water [8,17,18], only few studies have hitherto taken into consideration other sources that contribute towards the daily intake of fluoride [10,15,16]. The growing rates of globalisation and technological advancements have contributed to the increased consumption of a diverse range of commercially prepared ready-to-eat (RTE) infant products [19]. Globally, several studies have reported a varied level of fluoride concentration in RTE infant and children’s foods and/or drink products [19,20,21,22,23,24,25]. For example, a study from Iran [20] reported the mean fluoride content between 1.32–2.36 (standard deviation (SD) 0.1–0.3) μg F/g in collected samples of infant powdered milk products. Likewise, a study from Poland [25] reported a high level of fluoride content (0.35–1.14 ppm) in the samples of beverages for infants and young children. However, there is no similar study reporting fluoride concentration among commercially available infant RTE food products in Sydney, Australia. This study hypothesised that a high level of fluoride content is present in commercially available infant RTE food products available in Australian supermarkets. In view of this, the present study is aimed at addressing currently deficient, yet essential, knowledge by analysing fluoride content in a wide range of commercially available RTE products in Australia. The level of fluoride content in commercially available RTE food products is crucial for the revision or sustainability of Australian guidelines for fluoride concentration levels in RTE food products to achieve oral health goals.

## 2. Materials and Methods

In this study, 326 RTE infant solid and 49 liquid samples were randomly selected from health food/grocery shops and supermarkets across Sydney, Australia in 2017. These food samples were chosen to be representative of solid and liquid infant foods available in the markets of Sydney at the time. We have included the brand name, brief description of the food sample and its type, batch number, and product code of all collected samples being studied.

The fluoride content of a series of commercially available baby and infant food, beverage and formula products was correspondingly determined using potentiometry. In the experiment, an electrochemical cell consisting of a fluoride ion selective electrode (Orion Pacific Pty Ltd., Wallsend, NSW, Australia) and an Ag|AgCl reference electrode placed in 0.5 mL of a 0.1 M total ionic strength adjustment buffer (TISAB) containing either a liquid sample or a solid sample (preparation of the sample is described below). The voltage of the cell was repeatedly measured three times with a 10 s interval using a Hanna Instrument pH 211 microprocessor pH meter. Fluoride concentration in each sample was estimated based on an external calibration method.

In analysing a solid or a semi-solid sample, 1.0 g of a homogenised sample was placed in a petri dish, where micro diffusion was conducted to liberate the fluoride ions. In this process, a catalyst, hexamethyldisoloxane (acquired from Sigma Aldrich (Sydney, Australia), 99% *v*/*v*, 0.040 L) was initially added to a cooled perchloric acid solution (Sigma Aldrich; 1.0 mL, 5.8 M) and shaken for 3 min, before 1.0 mL of the mixture was added to the homogenised sample. The petri dish was then immediately covered by a lid that was pre-prepared with 5 well-separated drops of 0.50 M sodium hydroxide (Sigma Aldrich; 10 μL per drop) and sealed with Parafilm. The petri dish with its content was shaken at 600 rpm for 6 h on a Heidolph Unimax 1010 Rotary Shaker.

While the sample bases were discarded, the contents on the petri dish lid were quantitatively transferred to a borosilicate petri dish and left overnight in a desiccator to allow the fluoride trapped solution to dry to a white colour. An acetic acid and acetate buffer of pH 5.6 (100 μL) was added to each of the five white fluoride droplets to neutralise the solution. This solution was then mixed with TISAB for analysis in the electrochemical cell. Liquid samples (1.0 mL) were prepared directly for analysis without micro diffusion. 

The fluoride content of the collected samples for analyses was expressed in micrograms of fluoride per gram for solids (µg F/g) or millilitre for liquids (µg F/mL).

## 3. Results

All products were manufactured in Australia. The food type, mean (and its SD), median, and the range of fluoride concentration are presented in Table 1. Among the 375 collected samples, there were 326 RTE solid foods and 49 liquids.

### 3.1. Solid RTE Infant Foods

The overall median (range) fluoride content of the collected solid food samples was 0.16 (0.001–2.8) µg F/g, with meat + vegetable products showing the greatest variation (0.001 to 2.9 µg F/g), closely followed by vegetables alone (0.002 to 2.6 µg F/g). Under the meat + vegetable category, beef + vegetable samples were found to have the highest fluoride content (0.003 to 2.8 µg F/g), whereas lamb + vegetable samples showed the highest median (range) fluoride concentration of 0.74 (0.0711–1) µg F/g. Discretionary foods, including snacks and desserts, ranged from 0.003 to 1.6 µg F/g, while all other solids such as fruits, cereal, and formulas varied from 0.001 to 1.8 µg F/g. 

**Figure 1:**
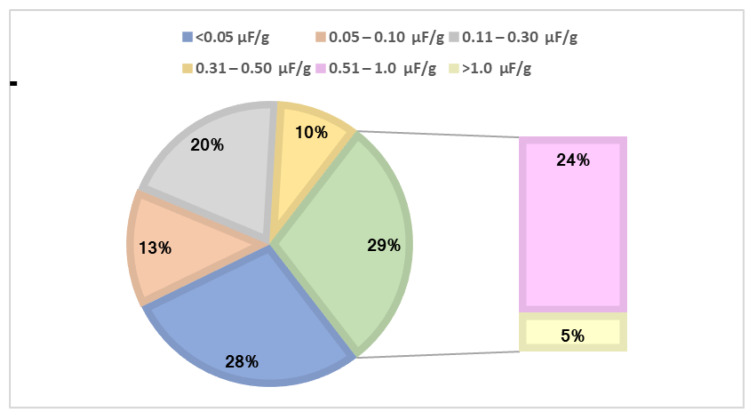
The percentage distribution of fluoride concentration in solid food samples (*n* = 326).

We have summarised the percentage distribution of fluoride content in solid food samples in Figure 1. Among the 326 solid RTE samples, 28% showed fluoride concentrations <0.05 µg F/g; 43% were in the 0.05–0.50 µg F/g range; 24% in the 0.51–1.0 µg F/g range; and 5% of samples were >1.0 µg F/g.

### 3.2. Liquid RTE Infant Foods

The overall median (range) fluoride concentration in liquid food samples was 0.020 (0.002–1.2) µg F/mL. Juices displayed the highest variation of fluoride content ranging from 0.004 to 1.2 µg F/mL. In contrast, milk and liquid formula samples were found to show the narrowest range of fluoride content 0.001 to 0.560 µg F/mL and 0.003 to 0.035 µg F/mL. Six samples of soy exhibited the highest mean (SD) fluoride content among liquids of 0.561 (0.40) µg F/mL.

**Figure 2:**
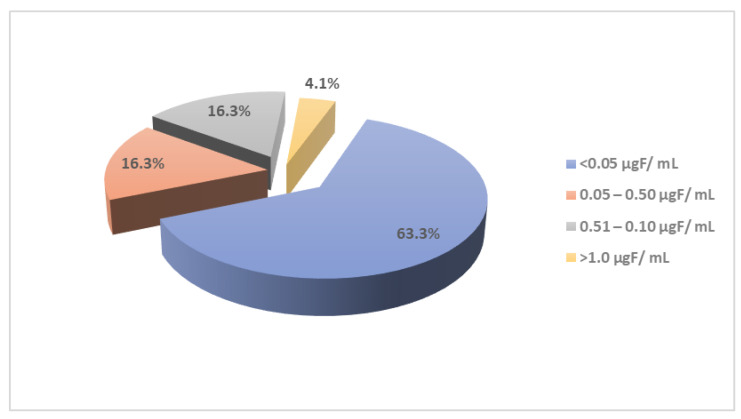
The percentage distribution of fluoride concentration in liquid food samples (*n* = 49).

Figure 2 shows the percentage distribution of fluoride content among liquid food samples. Based on 49 samples, 63.3% had concentrations <0.05 µg F/mL; 32.6% in the 0.05–1.0 µg F/mL range; and 4.1% > 1.0 µg F/mL.

### 3.3. Comparison of Fluoride Concentration in Formulas Targeting Different Age Groups

Table 2 tabulates the fluoride content of the 41 RTE foods and drinks by manufacturer-recommended age groups. An upward trend in the mean fluoride concentration of RTE foods and drinks is displayed with the suggested age of consumption from birth to 16 months onwards. However, there are some overlapped ranges of the fluoride concentration of RTE foods and drinks between age groups.

## 4. Discussion

This study is aimed at analysing fluoride content in a range of commercially available RTE products in Australia. A total of 375 RTE infant food and drinks samples was analysed for their fluoride content. A significant number of widely available RTE infant foods and drinks in the pharmacies and supermarkets of Sydney, Australia was selected and the fluoride level was determined using a potentiometric technique. The information on fluoride content in commercially available RTE products is crucial for the revision and/or sustainability of Australian guidelines in order to target at achieving oral health.

The general consensus of the recommended daily intake of fluoride for infants ranges from 0.05 mg/kg/day to 0.07 mg/kg/day [10]. However, for nine-month-old formula-fed infants, the recommended fluoride intake per day varies from 1.5 mg F/kg/day to 0.9 mg F/kg/day. These variations substantially depict the different assumptions arising from infant energy requirements, proportion of formula food consumption, and the amount of energy extracted from complementary foods. An adequate intake for infants under six months has not been recommended due to their dependency on breastfeeding and a lack of consumption of fluoridated tap water. However, the estimated upper limit of fluoride for infants aged 0–6 months with a 6-kg body weight is 1.2 mg/day and for infants aged 7–12 months with a 9-kg body weight, it is 1.8 mg/day [26].

In this study, the fluoride content in the analysed samples of RTE infant juices, milk, and liquid formulas showed a wide variation. However, these ranges are still within those for infant formulas and food requiring preparation before feeding. For example, during the preparation process, the final amount of fluoride available for consumption heavily depends on the fluoride content in the milk or water used for reconstitution. The median fluoride contents in the mixed meat products, including the vegetables + beef, chicken, lamb, and fish, were higher than the RTE infant formulas. In addition, the fluoride content in most of the infant formulas (46.3%, *n* = 19/49) was less than 0.05 µg F/g with only one (2.2%) of the infant formula showing a fluoride content higher than 1.00 µg F/g. 

### 4.1. Fluoride Content in Infant Formulas

We next compared the current study of infant formulas fluoride content to those reported in several recent studies. The mean fluoride content of 0.24 µg F/g in this study is lower than 0.49 µg F/g found in the infant formula samples of a previous Australian study [27], 1.73 µg F/g infant powdered milk reported in an Iranian study [20], and 0.31 µg F/L in a Thai study [21]. However, 0.24 µg F/g in the present study is higher at 0.02 µg F/g from United kingdom [19], 0.07 µg F/L from New Zealand [22], 0.22 µg F/mL from Brazil [23], 0.045 µg F/L from Malaysia [28], and 0.09 µg F/g from Japan [24] (Table S1).

The present study shows an upward trend in the mean fluoride content of RTE infant formulas with an increase in age. A similar trend was observed in the UK study, where fluoride contents were reported to increase from birth (0.059 µg F/g), four months (0.11 µg F/g), six months (0.14 µg F/g) to ten months (0.184 µg F/g) [19]. In contrast, a study from Japan [24] reported reduction in the fluoride content of RTE foods prepared with distilled and fluoridated water from birth to 6 months. However, the overlapping results were observed in the formula fluoride content for 12 months. The formula prepared from distilled water showed reduction in the fluoride content from birth, and the reported fluoride content was higher than 6 months. However, a significant increase was observed from birth to six months in the fluoride contents in specifically manufactured formulas for 12 months prepared with fluoridated water (Table S2). 

### 4.2. Fluoride Content in RTE Infant Food and Drinks

The range of fluoride content in various food categories reported by several recent studies is summarised in Table S3. In comparing the results obtained in the current study infant RTE liquid drinks to those in Table S3, a higher fluoride content variation (0.001–0.56 µg F/mL) was found in the cow milk and soymilk products [19]. Likewise, the fluoride concentration variation in the commercially available infants’ juices in the present study were higher (0.004–1.2 µg F/mL) in comparison to other studies [25,29,30] with the reported fluoride concentration, which was <0.30 µg F/g. 

With respect to RTE solid products in this study, the fluoride content variations are higher in comparison to the other studies, except those reported in Brazil [19,29,30]. The higher fluoride variation in the commercially available infant meat and chicken products in this study might be due to the addition of vegetables. Moreover, it might be due to the mechanical deboning processing techniques. Most meat products, especially chicken products ground into pulp using mechanical deboning processes, may contain a high concentration of fluoride content because these techniques leave behind residual bone and skin in the food [19,31]. A study from the UK reported the highest fluoride variation in commercially available meat products for infants (0.04–1.20 µg F/g), followed by vegetables (0.04–0.31 µg F/g), and chicken and turkey (0.07–0.27 µg F/g) [19]. However, a study from Japan reported their highest fluoride variation in vegetables (0.04–0.56 µg F/g), followed by cereal and meat [29].

Exclusive breastfeeding is recommended for infants until approximately six months of age, although some infants do rely on infant formulas. The mean fluoride content of the selected samples of infant RTE foods and liquid products appears to be lower than the upper limit for daily intake (0–6 months: 1.2 mg/day/6-kg body weight; 7–12 months: 1.8 mg/day/9-kg body weight) for infants recommended by the Department of Health of Australia [26]. However, these RTE foods substantially contribute to infant daily fluoride adequate intake. The daily fluoride ingestion by infants is not just determined by the fluoride concentration in the formulas and other food products, but also variation in infant dietary habits and intakes, and reconstitution of infant formulas either with distilled or fluoridated water may play a crucial role in disrupting the infant fluoride intake threshold. 

### 4.3. Study Limitations

The results of this study are based on the samples of a non-exhaustive list of commercially available infant RTE food and liquids from Sydney supermarkets and pharmacies, which limits the representation of the other products available across Australia. The results of the study are limited to the laboratory findings, while the reconstitutions of the similar products, with or without fluoridated water, has not been undertaken. In addition, the results are only applicable to the use of the RTE products as per manufacturers’ recommendations.

### 4.4. Implications and Recommendations

The findings of this study demonstrate a wide variation observed in some commercially available infant formulas for under six months. The results of this work may raise the awareness of parents regarding fluoride content in these products in an effort to avoid excessive daily fluoride intake. This will also allow parents to carefully monitor infant daily fluoride consumption through different food sources to minimise the chances that dental fluorosis may occur. The role of the health professional is crucial in disseminating the awareness of fluoride content in infant foods and drinks to parents and to provide guidance in terms of the appropriate timing of an introduction of these products to an infant’s diet. In addition, manufacturers should clearly label the fluoride content in all commercially available RTE products. The guidelines need to be developed to control the fluoride concentration in infant RTE formulas, aligned with the infant feeding guidelines. Moreover, additional studies are needed to focus on the actual intake of fluoride by infants through different food products, which will be essential in revising the fluoride content in manufactured RTE foods and updating the corresponding feeding guidelines for infants. In this way, consumers can monitor the fluoride content by preparing the formulas in fluoride-free water or limiting the quantity of a combination of fluoride rich food in the infant diet. Meanwhile, if necessary, manufacturers could potentially limit the fluoride content in RTE foods during the production process. 

## 5. Conclusions

In this study, we have observed a wide variation in the fluoride concentrations of RTE infant food and drinks, which may have arisen from the processing steps, different ingredients and their origins (including water). It is difficult to assess the contribution of RTE infant foods and drinks to an individual child’s daily fluoride intake, as manufacturers may use a variety of processing plants to manufacture RTE infant foods and drinks. Fluoride content, in most of the collected samples of RTE infant foods and drinks from Australian markets, appear to be high and may carry a risk of dental fluorosis. It is pertinent that parents receive appropriate information on the fluoride content of RTE infant food and drinks.

## Figures and Tables

**Table 1:** The fluoride content of 375 ready-to-eat (RTE) infant food and drinks samples.

Food/Drink Types	Number of Samples	F Concentration (µg/g or µg/mL)			95% CI
		Mean (SD)	Median	Range	
Solid RTE Foods					
Meat and vegetables					
Beef and vegetables	33	0.51 (0.53)	0.39	0.003–2.8	0.322–0.70
Chicken and vegetables	24	0.53 (0.50)	0.36	0.001–2.3	0.324–0.75
Lamb and vegetables	9	0.66 (0.33)	0.74	0.071–1.0	0.406–0.92
Fish and vegetables	6	0.31 (0.32)	0.21	0.004–0.79	−0.031–0.65
Discretionary foods					
Snacks	50	0.25 (0.38)	0.06	0.003–1.6	0.140–0.36
Desserts	42	0.36 (0.35)	0.23	0.004–1.4	0.249–0.47
Fruits	53	0.18 (0.23)	0.08	0.001–0.87	0.123–0.25
Vegetables	35	0.54 (0.71)	0.23	0.002–2.7	0.300–0.78
Cereals	34	0.42 (0.51)	0.11	0.001–1.8	0.239–0.59
Solid Formulas	40	0.27 (0.53)	0.12	0.010–1.2	0.163–0.38
Liquid RTE foods					
Milks	16	0.04 (0.14)	0.01	0.001–0.56	−0.030–0.12
Soy Milks	6	0.56 (0.40)	0.73	0.050–0.91	0.141–0.98
Juices	22	0.31 (0.44)	0.05	0.004–1.2	0.115–0.50
Liquid Formulas	5	0.03 (0.010)	0.03	0.003–0.035	0.163–0.38

RTE–Ready-to-eat, F–fluoride, g–Grams, µg–Microgram, L–Litre, mL–Millilitre, SD–Standard deviation, CI–Confidence Interval

**Table 2:** The fluoride concentrations of ready-to-eat infant formulas by manufacturer-specified age group.

Age Ranges Specified on Product	Number of Items	Fluoride Content (µg F/g or µg F/mL)		
		Mean	Median	Range
From Birth	19	0.24	0.12	0.013–1.2
>6 months	11	0.28	0.091	0.014–0.92
>12 months	08	0.29	0.17	0.010–0.80
≥16 months	03	0.34	0.13	0.040–0.84
Total	41	0.26	0.12	0.010–1.2

F–fluoride, g–Grams, µg–Microgram, L–Litre, mL–Millilitre.

## Data Availability

The data presented in this study are not publicly available due to privacy.

## Supplementary Materials

The following supporting information can be downloaded at: https://www.mdpi.com/article/10.3390/ijerph192114087/s1, Table S1: Summary of studies on fluoride concentration in infant formulas, Table S2: Summary of studies on fluoride content of infant formulas based on manufacturer-specified age group, Table S3: Summary of studies for the fluoride concentration in RTE foods and drinks.
