# Supplementary material for: Fluoride Content of Ready-to-Eat Infant Foods and Drinks in Australia
Source: Int J Environ Res Public Health. 2022 Oct 28;19(21):14087. doi: 10.3390/ijerph192114087 (PMC9655222; doi:10.3390/ijerph192114087)
Supplement: Supplementary file 1 [file ijerph-19-14087-s001.zip › ijerph-1962030-supplementary.pdf]

Supplementary Material

Table S1. Summary of studies on fluoride concentration in infant formulas.

Reference	Country	Sample Size	Fluoride content (μg F/g and μg F/mL or L)	
			**Range**	**Mean**
This work	Australia	45	0.014-1.1 (distilled water)	0.24
Maguire et al. (2011)	United Kingdom	147	0.01–0.03	0.02
Kaophun *et al.* (2018)	Thailand	17	0.24-0.30	0.31
Nohno *et al.* (2011)	Japan	22	0.039 - 0.13 (distilled water)<br>0.078 - 0.26 (0.13 μg F/ mL water)	0.09 (distilled water)<br>0.18 (0.13 μg F/ mL water)
Clifford (2009)	Australia	33	0.24–0.92	0.49
Nagata *et al.* (2016)	Brazil	15	0.04-1.2	0.22
Cressey (2010)	New Zealand	19	0.024-0.20	0.069
Mohd *et al.* (2020)	Malaysia	29	0.009 to 0.20 (deionised water)	0.045
Mahvi (2012)	Iran	12	1.0-2.2	1·7

F – fluoride, g – Grams, μg – Microgram, L – Litre, mL – Millilitre.

Table S2. Summary of studies on fluoride content of infant formulas based on manufacturer-specified age group.

Reference	Country	Specified Age	Fluoride content (μg F/g and μg F/mL)	
			**Mean**	**Range**
This study	Australia	From Birth	0.24	0.013-1.2
		>6 months	0.28	0.014-0.92
		>12 months	0.29	0.010-0.80
		≥16 months	0.34	0.040-0.84
Nohno *et al.* (2011)	Japan	From Birth	0.013 (distilled water)<br>0.026 (fluoridated water)	0.039–0.098 (distilled water)<br>0.078–0.20 (fluoridated water)
		From 6 months	0.011 (distilled water)<br>0.021 (fluoridated water)	0.087–0.092 (distilled water)<br>0.17–0.18 (fluoridated water)
		From 12 months	0.012 (distilled water)<br>0.023 (fluoridated water)	0.11–0.13 (distilled water)<br>0.21–0.26 (fluoridated water)
Maguire *et al.* (2011)	United Kingdom	From Birth	0.059	0.010–0.25
		From 4 months	0.11	0.020–0.50
		From 6 months	0.14	0.10–0.51
		10 months	0.18	0.06–1.2

F – fluoride, g – Grams, μg – Microgram, L – Litre, mL – Millilitre.

Table S3. Summary of studies for the fluoride concentration in RTE foods and drinks.

Reference	Fluoride content ranges (µg F/g and µg F/mL)									
	Country	Juices	Milk	Vegetables	Cereals	Fruits	Desserts	Meats	Fish	Chicken
This study	Australia	0.004-1.2	0.001-0.56	0.002-2.7	0.001-1.8	0.001-0.87	0.004-1.3	0.003-2.818 (beef + vegetables, and lamb + vegetables)	0.004-0.79 (fish and veg)	0.001-2.3 (chicken vegetables)
Maguire *et al.* (2011)	United Kingdom	0.05–0.15	0.01–0.03	0.04–0.31	–	0.02–0.19	0.03–0.38	0.04–1.20 (lamb, beef, pork)	0.11–0.23	0.07–0.27 chicken and turkey
Tomori *et al.* (2004)	Japan	0.14–0.18	–	0.04–0.56	0.06–0.44	–	–	0.02–0.18 (meat and fish)	–	–
Buzalaf *et al.* (2004)	Brazil	0.01–0.30	–	–	0.2–7.8	–	–	–	–	–
Opydo-Szymaczek and Opydo (2010)	Poland	0.01- 0.29	–	–	–	–	–	–	–	–

F – fluoride, g – Grams, µg – Microgram, L – Litre, mL – Millilitre.
